# Identification of specific reference gene for normalization of RT-qPCR data in rhythmic gene expression studies of the effect of developmental hormone antagonist in postembryonic development in *Bombyx mori*

**DOI:** 10.3389/finsc.2024.1362473

**Published:** 2024-06-28

**Authors:** Minurani Dalai, Anita Jagota

**Affiliations:** Neurobiology and Molecular Chronobiology Laboratory, Department of Animal Biology, School of Life Sciences, University of Hyderabad, Hyderabad, Telangana, India

**Keywords:** daily rhythms, *Bombyx mori*, CNS, RT-qPCR, reference gene, PED, Precocene 1 and testosterone

## Abstract

*Bombyx mori* is a lepidopteran holometabolous insect with distinct developmental stages: egg, larvae, pupae, and adult. The lepidopteran insect undergoes major modifications in the central nervous system (CNS) so as to adapt to the lifestyle of these distinct stages with specific habitats and functions from voraciously feeding larval stages to flying reproductive adults via dormant pupal stages. Such transitions are linked to transcriptional, epigenetic, and translational complexities. Therefore, studying rhythmic gene expression in CNS of various developmental stages and the effects of antagonists on developmental hormones requires a very stable reference gene (RG). To facilitate rhythmic gene expression studies using reverse transcription quantitative polymerase chain reaction (RT-qPCR) in *B. mori* and the effect of developmental hormone juvenile hormone (JH) and 20-hydroxy ecdysone hormone (20 HE), antagonists Precocene 1 and testosterone, respectively, were used. Eight candidate RGs, namely, *Translational initiation factor 3 subunit 4* (TI3S4), *Translational initiation factor 3 subunit 5* (TI3S5), *Ribosomal protein subunit 7* (RPs7), *TATA-binding protein association factor* (TAF13), *Translational initiation factor 4 A* (TI4A), *Ribosomal protein* (RPL32), *Elongation factor* 1 (EF1), and *Arginine kinase* (AK), were assessed in the CNS of *B. mori*. The postembryonic developmental (PED) stages used were the fifth late larval instar, early pupa, mid pupa, late pupa, and adult. The assessments were done at four different time points, Zeitgeber time (ZT) 0, 6, 12, and 18, to find stability towards 24-h rhythmic expression. RefFinder, geNorm, and Ct value analysis were performed. RefFinder and geNORM studies suggested stability order as TI3S4 > TI3S5 > RPs7, but Ct value evaluation showed stability order as TI3S5 > TI3S4 > RPs7. We therefore demonstrated that TI3S4, TI3S5, and RPs7 can be used as RG in various PED stages in CNS of *B. mori* (Strain: CB-hybrid, PM×CSR2) towards studies with effects of JH and 20 HE antagonists.

## Introduction

The silkworm, *Bombyx mori*, is an economically important holometabolous lepidopteran insect with distinct developmental stages such as larva, pupa, and adult. It undergoes complex morphological and physiological changes during the metamorphosis (1). These changes are linked to several modifications in the central nervous system (CNS) and associated peripheral changes (2). The molecular mechanism of such alterations can be studied by gene expression analysis with the help of reverse transcription quantitative polymerase chain reaction (RT-qPCR). It is most widely used for its accuracy, high sensitivity, reliability, reproducibility, and cost-effectiveness (3). However, the selection of an appropriate housekeeping gene/reference gene (RG) for data normalization of biological samples is the prerequisite in gene expression studies (4). An ideal reference gene should be consistently expressed, with its stability and expression being independent of any experimental conditions. RG should show a moderate threshold cycle (5–7). Generally, 18S ribosomal RNA (18s rRNA), beta-actin (β-ACT), and glyceraldehyde, 3-phosphate dehydrogenase (GAPDH) are used as RGs, since they are involved in the basic biochemical metabolic processes of the organism, linked to components of the cytoskeleton, and stably expressed in different cells (8, 9).

Several reports have been linked to the ineffectiveness and variable expression of these commonly used RGs under different experimental conditions (8, 10). Reports have shown unstable expression of GAPDH and 18s rRNA in various stages and different tissues of *B. mori* (11). In *B. mori* (Strain: Dazao), certain genes involved in basic biochemical processes show variation in different stages, tissues, cell proliferation, and development, which can be a limiting factor in RT-qPCR data normalization (1, 11). The unstable expression can reflect discrepancies in the data; hence, studies by earlier workers have suggested that screening and validation of RGs under specific experimental conditions is necessary (8). TI3S4 and TI3S5 were reported to be stable RGs in *B. mori* (Dazao) for early 5th instar larvae in different tissues such as silk gland, testes, ovaries, fat body, midgut, integument, hemocytes, and Malpighian tubule (12, 13). Furthermore, *RP49/RPs7* and GAPDH were reported to be stable RGs in egg, larva, pupa, and adult stage, and GAPDH, EF1, and *RP49/RPs7* showed stable expression in head, midgut, ovary, testis, fat body, Malpighian tubules, silk gland, and epidermis of *B. mori* (Dazao) (14). Most ribosomal genes and genes involved in eukaryotic translation initiation function were linked to uniform expression in different developmental stages of Chinese *B. mori* (Strain: Dazao) (1, 15).

The aim of this study is to find suitable RGs for *B. mori* (Strain: CB-hybrid, PM×CSR2) in different experimental variables such as developmental stages, i.e., late larval instar (LLI), early pupa (EP), mid pupa (MP), late pupa (LP), and adult after 24 h of eclosion (A). Furthermore, the effects of developmental hormone JH antagonist (Precocene 1) and 20HE antagonist (testosterone) were also studied on candidate RGs. The effect of Precocene 1 has been reported to be stage specific in different holometabolous insects. In *Chrysomya megacephala*, a dose of 200 µg showed 10% mortality, whereas with 300 µg, no mortality was observed (16). Similarly, in *Eurygaster integriceps* (hemimetabolous), even with the highest dose, no mortality was observed (17). Furthermore, high mortality with lower doses was reported for *Spodoptera littoralis* and *Euprepocnemis plorans plorans* (18, 19). Eight candidate RGs were selected based on their stable expression in various experimental conditions in different lepidopteran insects such as Chinese *B. mori* (Strain: Dazao), *Spodoptera exigua*, *Plutella xylostella*, *Cryptophlebia peltastica*, *Thaumatotibia leucotreta*, *Cydia pomonella*, and hymenopteran (*Apis mellifera*) and dipteran (*Drosophila suzukii*). These RGs were *Translational initiation factor 3 subunit 4* (TI3S4) (12), *Translational initiation factor 3 subunit 5* (TI3S5) (12), *Ribosomal protein subunit 7* (RPs7) (14), *TATA-binding protein association factor* (TAF13) (20), *Translational initiation factor 4 A* (TI4A) (21), *Ribosomal protein* (RPL32) (22), *Elongation factor* 1 (EF1) (23), and *Arginine kinase* (AK) (24). The gene expression analysis is studied for the stability of these in various time points of a 24-h light–dark cycle so as to use selected RGs for rhythmic gene expression in CNS of variable developmental stages upon treatment with developmental hormone antagonists in *B. mori*. Furthermore, we evaluated two doses of Precocene 1, namely, 200 and 300 µg, per insect to eliminate any dose-specific mortality.

## Methodology

### Insect maintenance

The insects were obtained from the local sericulture department and maintained at LD 12:12 photoperiod, with a temperature of 26 ± 1°C and a relative humidity of 70% ± 5%. Larvae were fed with fresh mulberry leaves provided *ad libitum*. Various developmental stages such as LLI, EP, MP, LP, and A were used in the study. Adult insects were obtained after 24 h of post eclosion; both male and female insects were included in the study. The insects were ice narcotized and the CNS was dissected out in Insect Ringer’s solution (25). The samples were collected at four different time points, i.e., Zeitgeber time (ZT) 0, 6, 12, and 18.

### Antagonist treatment

#### JH antagonist (Precocene 1)

Two doses of Precocene 1 (CAS-17598–02-6, 1 g/mL, Sigma), 200 µg (Dose 1) and 300 µg (Dose 2), were used in this study. A solution (300 µg/µL) of Precocene 1 was prepared by dissolving 57 µL of Precocene 1 in 200 µL of acetone (16, 26). For larvae, 0.67 and 1 µL of this solution were topically applied to provide 200 and 300 µg/insect, respectively (17), whereas for pupae and adults, 0.67 and 1 µL of Precocene 1 solution (300 µg/µL) were further diluted with insect Ringers solution to 5 µL and injected per insect at the third thoracic segment with the help of Hamilton syringes (27).

#### 20HE antagonist (testosterone)

A testosterone (CAS-58–22-05 g, HPLC ≥99.0%, Sigma) stock solution (1 mg/mL) was prepared by dissolving it in acetone for testosterone treatment (TT). All stages were treated with 1 mg of testosterone/kg body weight of insect. Larvae, pupae, and adults weighed approximately 2.5, 1.5, and 1.1 g, respectively. For larvae, ~2.5 µL of testosterone stock solution was applied on a square inch of leaf, and larvae were allowed to eat it completely (28). Larvae were not fed prior to dosing in order to ensure complete consumption of the leaf. Pupae and adults were injected with ~1.5 and 1.1 µL of the TT stock solutions after diluting to 5 µL with Insect Ringers solution respectively on the 3rd thoracic segment with the help of Hamilton syringe (27).

### RNA extraction and cDNA synthesis

Total RNA was extracted using TRI reagent (Sigma) following the manufacturer’s protocol and was dissolved in nuclease-free water. The concentration and purity of RNA were quantified by measuring optical density (OD) at 260 and 280 nm using a Nanodrop spectrophotometer (Thermo Fisher). cDNA synthesis was performed by using the Bioline SensiFAST cDNA synthesis kit by following the manufacturer’s protocol. For RT-qPCR analysis, 4 μl of cDNA (diluted to a concentration of 1:20 in nuclease-free water) was used (29).

### Reference gene selection and primer design

Eight candidate RGs were selected (**Table 1**). TI3S4, TI3S5, and TI4A primers were used as per Wang et al. (12). *B. mori* gene-specific primers were designed for RGs AK, RPs7, RPL32, TAF13, and EF1 by using genome sequencing data (KAIKO Database) available online. The primary design was done by the PRIMER QUEST software IDT (30).

**Table 1:** Various primers used for RT-qPCR.

S. no.	Gene	NCBI accession number	Primers	Amplicon size (bp)
1	TI3S4	DQ443289	F:ACTTCAAGTTCAGGGCAGAT R:TTAATTGTTTTGTGGAGGCT	110
2	TI3S5	NM_001047063	F:ATTGCAGCTCGCACATTC R:AGTTGGAGTTGGGTCTTGAT	126
3	TI4A	DQ443290	F:TTCGTACTGGCTCTTCTCGT R:CAAAGTTGATAGCAATTCCCT	174
4	RPs7		F:GCCTAAGCCCAGCCACAAA R:CCGTCCAACTTGACCCTGATG	147
5	EF1		F:ACGGAAGTGACTGTTTGAGCA R:GACGTGTCCGATGACGACAATG	161
6	RPL 32		F:CAGGCGGTTCAAGGGTCAATAC R:CACGATCAGCTTCCGCTTGTTC	199
7	TAF 13		F:GGTGGAACTACATCTGGTCGT R:CCAACTTCCATTGCCCTATGTG	164
8	AK		F:GCTCCAGGGATCCGACTCTAA R:CCAAGTTCTCGACACCCGATTG	134

1, 2, 3 as per Wang et al. (12).

4, 5, 6, 7, 8 were designed using the KAIKO database for *B. mori*.

### Reverse transcription quantitative PCR

The RT-qPCR reactions were carried out by the SYBR GREEN detection method in StepOne plus Applied Biosystems, Foster, USA. Thermo Fisher Power SYBR GREEN was used according to the manufacturer’s protocol (30). The cycling conditions consisted of 10 min of holding stage at 95°C, and the cycling stage consisted of 40 cycles of 95°C for 15 s, 60°C for 1 min, followed by one cycle of 95°C for 15 s, 60°C for 1 min, and 95°C for 15 s. The PCR specificity was monitored with melt curve analysis with the help of StepOne software v2.0.2. The specificity and reliability of the primers were validated by melt curves, and each melt curve showed a single sharp peak, suggesting a single gene amplification (**Supplementary Figure 1**). The cycle threshold (Ct), was determined by a log linear plot of fluorescent signal versus the thermal cycle numbers and is inversely correlated with the amount of template in the reaction (31).

### Data processing

The mean Ct values were calculated based on three biological replicates (*n* = 3, each sample contained 5 pooled CNS). In order to validate the stability of these eight genes and to identify the stable RG, RefFinder was used. RefFinder is a web-based comprehensive tool (http://www.ciidirsinaloa.com.mx/RefFinder-master/) that contains four universal analysis programs, i.e., comparative ΔCt method, NormFinder, geNORM, and BestKeeper (6, 32). Additionally, an online-based algorithmic tool, geNorm (Biogazelle qBase+), was used separately for further validation of RGs (33). The geNORM tool assessed and suggests the number of RGs needed to standardize gene expression and their stability in a given experimental condition. All the Ct values were collected from RT-qPCR with the help of StepOne software v2.0.2, and these values were used as input data for geNorm (Biogazelle qBase+) software; furthermore, mean Ct calculation was done with Microsoft Excel. As a comprehensive web-based platform, RefFinder integrated the results from geNORM, BestKeeper, NormFinder, and the ΔCt method and ranked the RGs. The graphs were plotted using Graph Pad Prism 7.0 (29).

## Results

### Specificity of primer sets and expression stability

For gene-specific primers, specificity was confirmed via NCBI BLAST; furthermore, the amplification specificity was validated by single sharp melt curve peaks (**Supplementary Figure 1**). TI3S4, TI3S5, and TI4A primers were used from a previous study in *B. mori* Chinese silkworm strain (Strain: Dazao) (12).

#### Ct values and its variation in candidate reference genes

**Figure 1:**
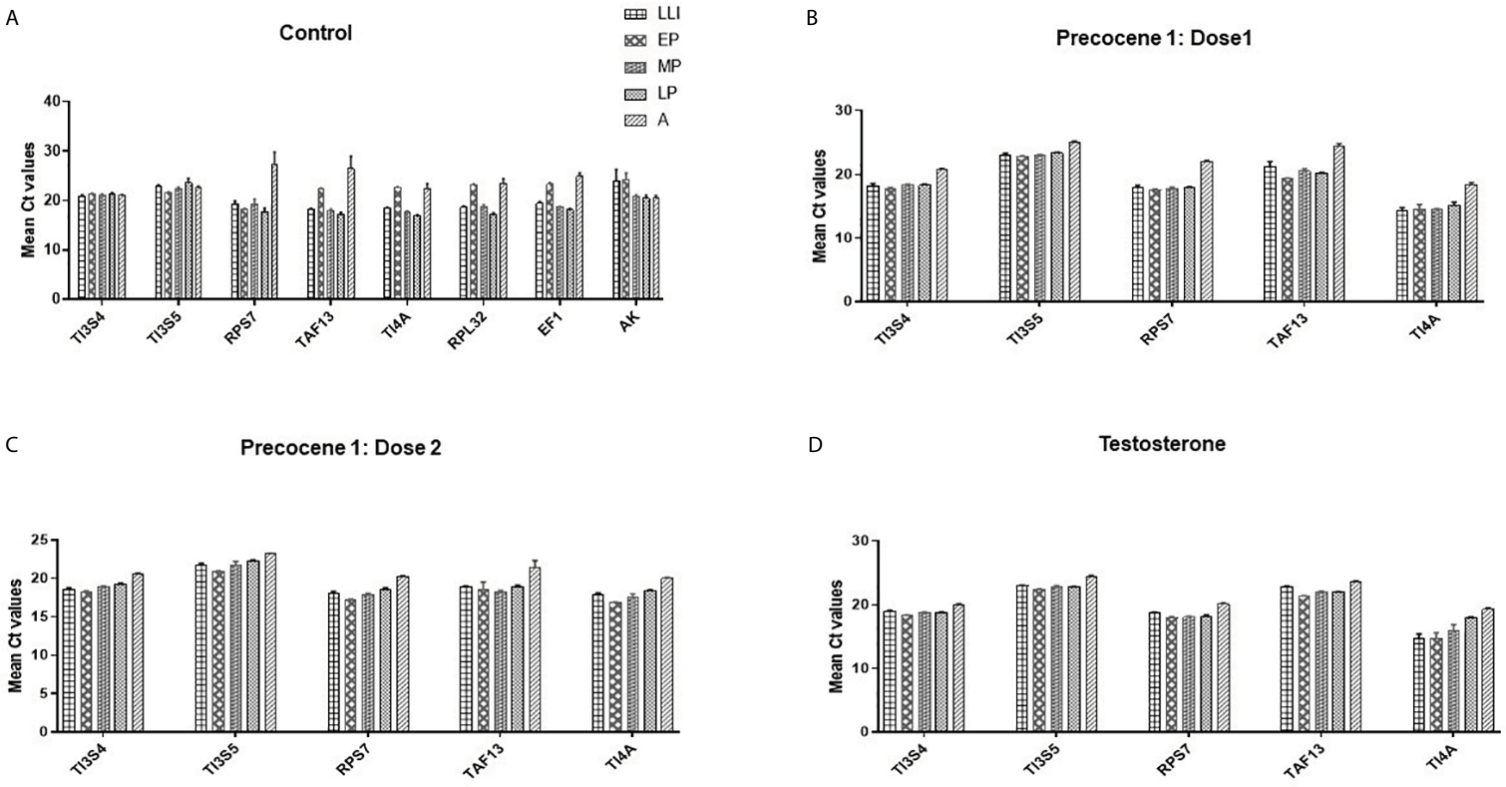
Mean Ct value analysis of the expression stability of selected reference genes in different parameters. **(A)** Control: TI3S4, TI3S5, and RPs7 showed the most stable expression in all the stages followed by TAF13, TI41, RPL32, EF1, and AK; **(B)** Precocene 1–Dose 1: 200 µg treatment; **(C)** Precocene 1–Dose 2: 300 µg treatment; **(D)** testosterone treatment: TI3S4, TI3S5, and RPs7 showed the most stable expression across the stages followed by TAF13 and TI4A in various PED stages.

The Ct values were converted to mean values for the analysis using Ct value analysis tools. Ct values for TI3S4, TAF13, and RPs7 were observed as 0.43, 1.07, and 1.17, respectively. Based on the Ct value analysis, the order of stability in different developmental stages and time points is TI3S4 > TAF13 > RPs7 > RPL32 > TI4A > TI3S5 > AK > EF1 (**Figure 1A**). It was observed in the control group that AK, EF1, and RPL32 are showing altered expression in different developmental stages and thus cannot be considered as RGs for further studies. No significant difference was observed in control and vehicle controls in all the stages and time points studied.

#### Analysis of expression stability and ranking of candidate reference gene during developmental stages by RefFinder

**Figure 2:**
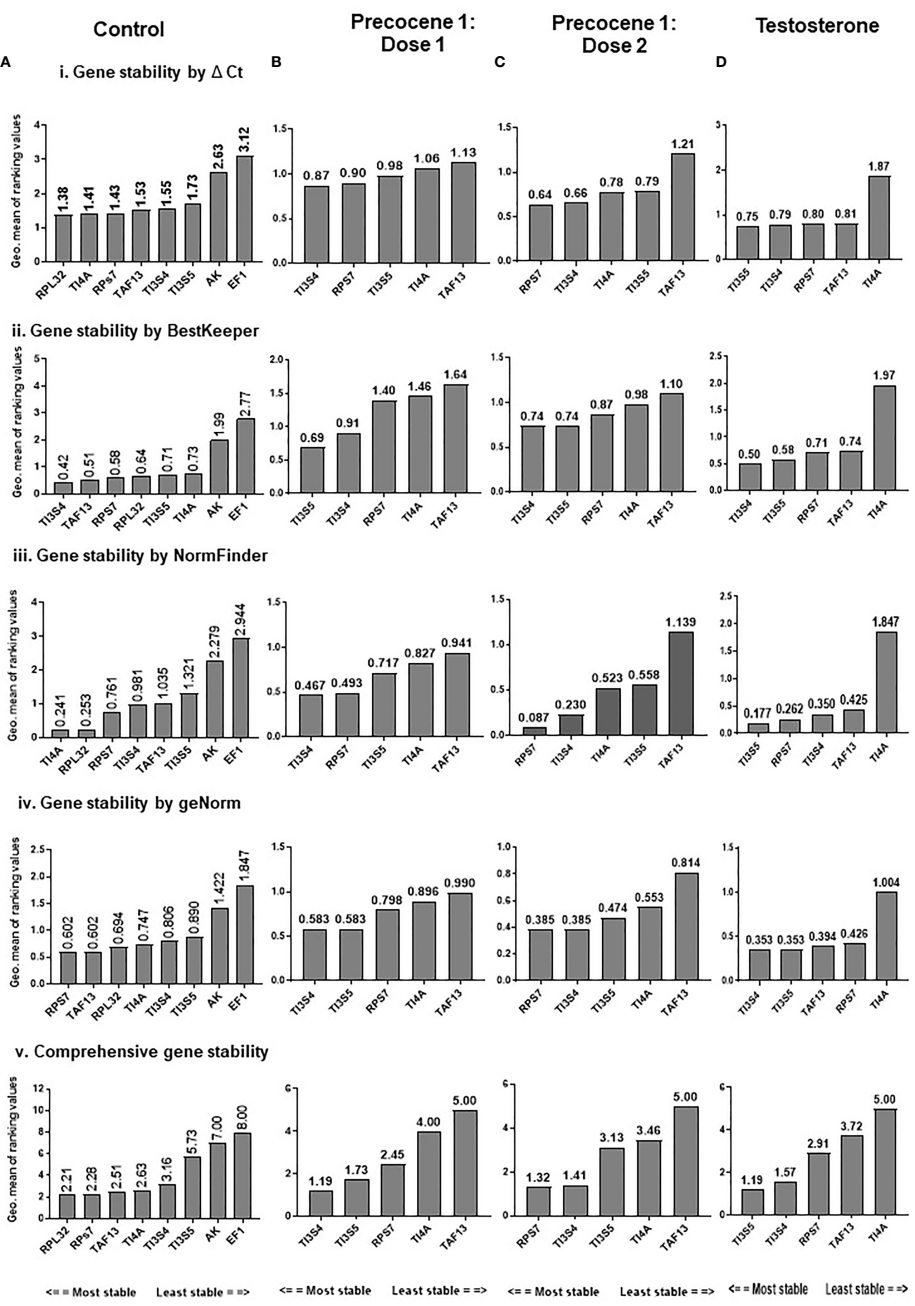
RefFinder analysis of candidate reference genes across the developmental stages of *B mori*; RefFinder integrates the results from the ΔCt method, BestKeeper, NormFinder, and geNORM and ranking the RGs based on their stability. (i) Gene stability ranking by ΔCt analysis; (ii) gene stability ranking order by BestKeepr; (iii) gene stability ranking order by NormFinder; (iv) gene stability ranking order by geNORM; (v) comprehensive ranking order of RGs by RefFinder. **(A)** Control, **(B)** Precocene 1–Dose 1: 200 µg treatment, **(C)** Precocene 1–Dose 2: 300 µg treatment, and **(D)** testosterone treatment. The genes are present with the stability decreasing from left to right.

The expression stability of eight candidate RGs was analyzed using RefFinder and the geNORM tool separately. RefFinder analysis was done for each developmental stage, and each program generated its individual ranking order (**Supplementary Figure 2A**). Additionally, a comprehensive analysis over different developmental stages showed RPL32, RPs7, and TI4A as the most stable RGs, and the stability ranking order was RPL32 > RPs7 > TAF13 > TI4A > TI3S4 > TI3S5 > AK > EF1 (**Figure 2A**).

#### Analysis of overall expression stability by the geNORM tool across developmental stages

**Figure 3:**
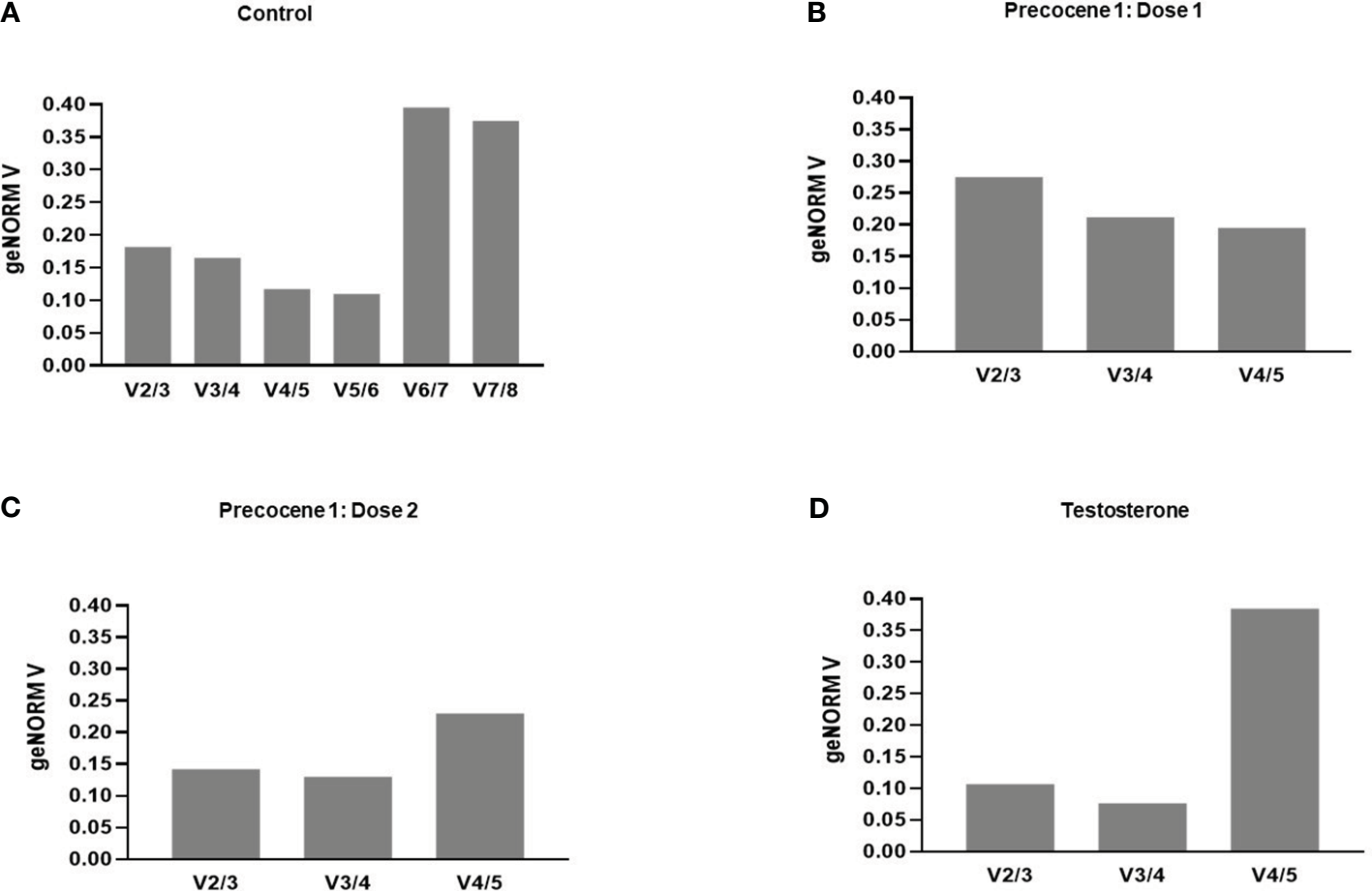
Pairwise variation (V) of the candidate reference genes by *B mori* by the geNorm algorithm (Biogazelle qBase+). Vn/n+1<0.15 suggests that “n” number of optimum RGs are required for the respective experimental **(A)** control, **(B)** Precocene 1–Dose 1: 200 µg treatment, **(C)** Precocene 1–Dose 2: 300 µg treatment, and **(D)** testosterone treatment.

Gene stability was calculated using the geNorm algorithm (Biogazelle qBase+) tool following Vandesompele et al. (34) and Hellemans et al. (33). The candidate RGs were ranked depending on their expression in four time points and PED stages. TAF13, TI3S5, RPs7, RPL32, TI4A, and TI3S4 showed an M value of less than 1.5, which indicates their stable expression in the provided experimental variation. On the other hand, RPL32, AK, and EF1 were considered as the least stable genes with M values of 2.01, 2.669, and 3.139, respectively (**Table 2**). The average pairwise variations V4/5 and V5/6 were below 0.15, suggesting that a maximum of five appropriate genes can act as potential RGs for qRT-PCR analysis (**Figure 3A**). The genes with the lowest M value were used for further validation under antagonist treatments (**Table 2**). Thus, stability ranking was established as TI4A > TI3S5 > TI3S4 > TAF13 > RPs7 > RPL32 > AK > EF1.

**Table 2:** Gene stability values (M values) of reference genes in different experimental conditions in *Bombyx mori* generated by the geNorm algorithm (Biogazelle qBase+).

Experimental factors	Genes							
	TI3S5	TI3S4	RPS7	TAF13	TI4A	RPL32	AK	EF1
**Control**	1.529	1.586	1.458	1.522	1.426	2.01	2.669	3.139
**Precocene 1–Dose 1 treatment**	0.922	0.8	0.852	1.099	1.017	–	–	–
**Precocene 1–Dose 2 treatment**	0.740	0.599	0.578	1.194	0.716	–	–	–
**Testosterone treatment**	0.7	0.737	0.732	0.776	1.93	–	–	–

#### Analysis of expression stability and ranking of candidate reference gene under antagonist treatment

##### Ct values and ranking of candidate reference genes

Based on the Ct difference in PED stages, the order of RGs’ stability in different developmental stages and Precocene 1 dose 200 µg/insect treatment showed that the range of expression of candidate RGs is TI3S5 > TI3S4 > TI4A > RPs7 > TAF13 (**Figure 1B**). However, in Precocene 1 dose 300 µg/insect and TT, the Ct values ranking was found as TI3S5 > TI3S4 > RPs7 > TAF13 > TI4A (**Figure 1C**). This suggested that TI3S5, TI3S4, and RPs7 can be considered as stable RGs under different developmental hormone antagonistic treatments (**Figure 1D**).

##### RefFinder analysis and ranking

RefFinder analysis was done under antagonist treatment in different developmental stages. In each stage ΔCt, BestKeeper, NormFinder and geNORM analysis showed differential ranking of RGs (**Supplementary Figure 2**). The recommended comprehensive ranking in the Precocene 1–200 µg treatment was TI3S4 > TI3S5 > RPs7 > TI4A > TAF13 and that in the Precocene 1–300 µg treatment was RPs7 > TI3S4 > TI3S5 > TI4A > TAF13. However, TT showed Ct value ranking as TI3S5 > TI3S4 > RPs7 > TAF13 > TI4A (**Figures 2B–D**).

##### geNORM tool analysis

Evaluation of RGs with the geNORM tool was carried out using the treatments. The treatment with Precocene 1 Dose 1 showed lower M values for TI3S4, RPs7, and TI3S5, i.e., 0.8, 0.852, and 0.922, respectively, than TAF13 and TI4A. However, with Precocene 1 Dose 2, the M values of TI4A, RPs7, and TI3S4 were the lowest, suggesting higher stability compared to TI3S5 and TAF13 (**Table 2**). Thus, the stability ranking in Precocene 1 Dose 1 was TI3S4 > RPs7 > TI3S5 > TI4A > TAF13 and that in Precocene 1 Dose 2 was RPs7 > TI3S4 > TI4A > TI3S5 > TAF13. With TT, TI3S5, RPs7, TI3S4, and TAF13 showed M values of 0.7, 0.737, 0.732, and 0.776, respectively, whereas TI4A showed a higher M value of 1.93. Therefore, the ranking stability was TI3S5 > RPs7 > TI3S4 > TAF13 > TI4A (**Table 2**). In Precocene 1 Dose 1, no pairwise variation was below 0.15, whereas in Precocene 1 Dose 2 and with TT, both V2/3 and V3/4 were below 0.15, suggesting that a maximum of three potential RGs can be used in these antagonist treatments (**Figures 3B–D**).

## Discussion

As a prerequisite towards the selection of an appropriate RG for understanding the effect of antagonists of insect developmental hormones on rhythmic gene expression in various PED stages in CNS of *B. mori*, we studied the expression stability of eight candidate RGs.

The RefFinder tool gives the stability ranking of each candidate gene by using various programs such as ΔCt, BestKeeper, NormFinder, and geNORM. The ranking orders are based on different statistical endpoints and algorithms, and the weight of each gene is determined by calculating the geometric mean (32, 35). Furthermore, the geNORM (Biogazelle qBase+) tool was used to validate the results through the calculation of pairwise variation value (Vn/n+1); a value of less than 0.15 indicates the optimum number of RGs for qRT-PCR, and it also provides a stability value (M) for the candidate genes (10, 23) (**Figure 3**, **Table 2**). Most of the ranking analyses obtained with different programs were consistent, suggesting the reliability of the results.

The stability of Ct values is crucial in determining the reliability of an RG, which is inversely proportional to the gene expression. The Ct values of candidate RGs varied from 16.92 to 27.43. The Ct values for RPL32 and RPs7 were found to be approximately 16, suggesting the optimum expression of these genes (36). In hemimetabolous, Thysanoptera insect *Megalurothrips usitatus* RPL and RPs were reported to be the most stable RGs in different developmental stages and under the treatment of insecticides (35). Furthermore, in another hemimetabolous, hemipteran insect *Eocanthecona furcellata* and holometabolous lepidopteran insect *Spodoptera frugiperda*, RPL32 and RPL13 along with some other RGs were reported to be the most stable RGs in different developmental stages (37, 38). In the current study, according to RefFinder and Ct analysis in the non-treated group, RPL32 showed a quite stable expression in different developmental stages of *B. mori*, but a separate analysis with geNORM showed it to be a fairly less stable expressing gene in developmental stages; however, RPs7 stability was consistent throughout the developmental stages. *RP 49/RPs7* was identified as the most stable RG for developmental studies in *A. mellifera* (20). Ribosomal proteins are responsible for ribosomal assembly and protein translation, contributing towards cell development and has been found to be the most stable gene for RT-qPCR studies (38). Few studies by the Qing-You Xia group have demonstrated that TI3S4, TI4A, and TI3S4 are stable RGs in early 5th instar larvae, 60 h after wandering (a stage between 5th late larval instar and early pupa), pupa, and adult *B. mori* insects (12). Additionally, TI3S4 and TI3S5 were observed to be stable RGs in various tissues of *B. mori*, which include silk glands, testes, ovaries, fat body, midgut, integument, hemocytes, and Malpighian tubules, specifically in early 5th instar larvae (13). However, other workers reported *RP49/RPs7* and GAPDH as stable RGs in different stages of Chinese *B. mori* development, including egg, larva, pupa, and adult stages (14). Furthermore, GAPDH, EF1, and *RP49/RPs7* were found to exhibit stable expression in various tissues of *B. mori*, such as the head, midgut, ovary, testis, fat body, Malpighian tubules, silk glands, head, and epidermis. A global transcriptome analysis conducted by Wang et al. (1) revealed that most ribosomal genes and genes involved in eukaryotic translation initiation function exhibit uniform expression across different developmental stages of *B. mori.*

Various studies of Chinese *B. mori* have consistently shown that the expression levels of multiple candidate RGs are influenced by specific experimental conditions, thus indicating that there is no universally applicable RG that can reliably serve under different experimental conditions. Hence, it is strongly recommended by few researchers to conduct a thorough validation and select custom RGs for any provided experimental conditions, even while working within the same species (15, 39). It is also suggested to use multiple RGs for the normalization of data; however, some researchers have reported the use of single RGs for normalization in insecticide treatment, developmental stage, and light conditions, and in different temperature conditions (34, 35, 40).

We report TI3S4, TI3S5, and RPs7 to be the most stable RGs in CNS under various experimental conditions such as different PED stages, different time points, and under two developmental hormone antagonist treatments in Indian *B. mori* (Strain: CB-hybrid, PM×CSR2). Another candidate RG, RPL32, can be used for gene expression studies in *B. mori* PED stages at various time points; however, it was not stable under developmental hormone antagonist treatment.

The RGs identified here will be used to study various clock gene expressions in CNS in different PED stages of *B. mori* at various ZTs to help understand the role of antagonists Precocene 1 and testosterone for developmental hormone JH and ecdysone in circadian function.

## Data availability statement

The datasets presented in this study can be found in online repositories. The names of the repository/repositories and accession number(s) can be found in the article/**Supplementary Material**.

## Ethics statement

The manuscript presents research on animals that do not require ethical approval for their study.

## Author contributions

MD: Conceptualization, Data curation, Formal Analysis, Methodology, Writing – original draft. AJ: Conceptualization, Formal Analysis, Funding acquisition, Investigation, Methodology, Project administration, Resources, Software, Supervision, Validation, Visualization, Writing – review & editing.

## Supplementary material

Supplementary Figure 1 Melt curves of selected reference genes showing single amplification.

Supplementary Figure 2 RefFinder analysis of candidate reference genes in different developmental stages of *B. mori*. (i) Gene stability ranking by ΔCt analysis; (ii) gene stability ranking order by BestKeepr; (iii) gene stability ranking order by NormFinder; (iv) gene stability ranking order by geNORM; and (v) comprehensive ranking order of RGs by RefFinder. **(A)** Control, **(B)** Precocene 1–Dose 1: 200 µg treatment, **(C)** Precocene 1–Dose 2: 300 µg treatment, and **(D)** testosterone treatment.
