# Supplementary figures and images for: Identification of specific reference gene for normalization of RT-qPCR data in rhythmic gene expression studies of the effect of developmental hormone antagonist in postembryonic development in Bombyx mori
Source: Front Insect Sci. 2024 Jun 28;4:1362473. doi: 10.3389/finsc.2024.1362473 (PMC11239437; doi:10.3389/finsc.2024.1362473)

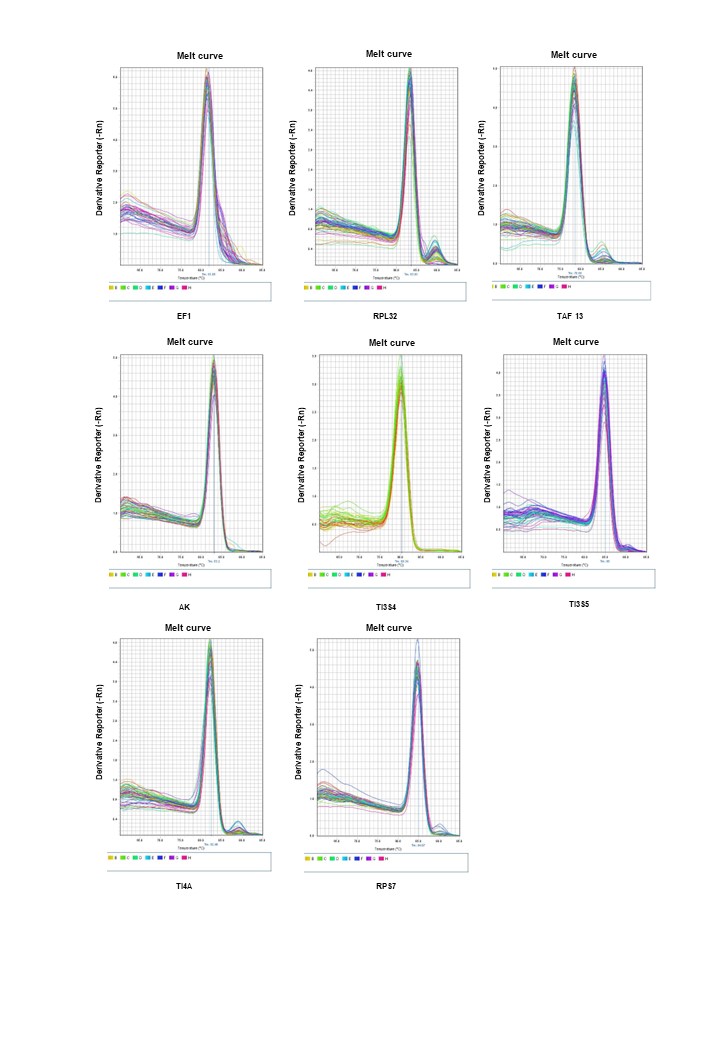

Supplement: Supplementary Figure 1 — Melt curves of selected reference genes showing single amplification. [file Image_1.jpeg]

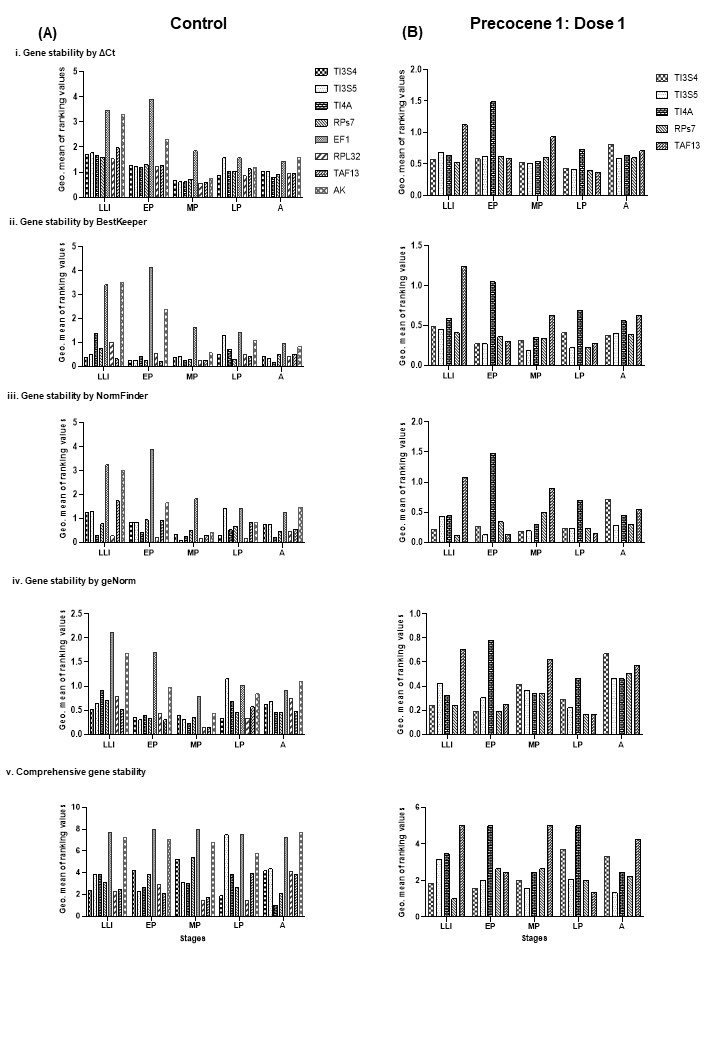

Supplement: Supplementary Figure 2 — RefFinder analysis of candidate reference genes in different developmental stages of B. mori. (i) Gene stability ranking by ΔCt analysis; (ii) gene stability ranking order by BestKeepr; (iii) gene stability ranking order by NormFinder; (iv) gene stability ranking order by geNORM; and (v) comprehensive ranking order of RGs by RefFinder. (A) Control, (B) Precocene 1–Dose 1: 200 µg treatment, (C) Precocene 1–Dose 2: 300 µg treatment, and (D) testosterone treatment. [file Image_2.jpeg]

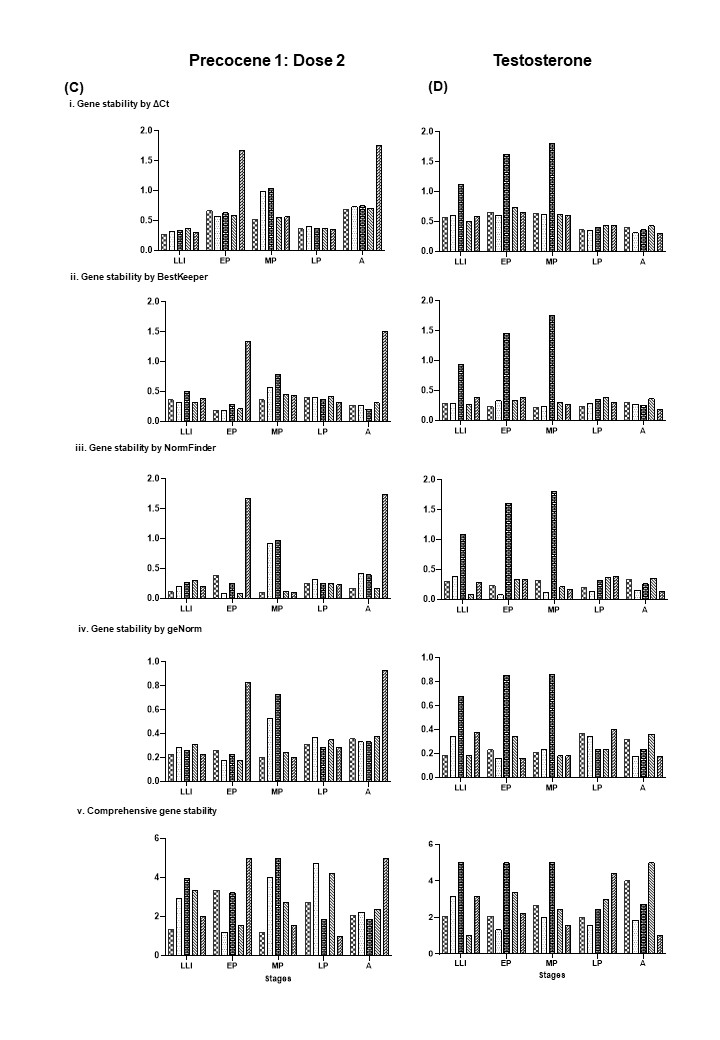

Supplement: Supplementary file 3 [file Image_3.jpeg]
